# Inhibition of phospholipase D promotes neurological function recovery and reduces neuroinflammation after spinal cord injury in mice

**DOI:** 10.3389/fncel.2024.1352630

**Published:** 2024-03-20

**Authors:** Han Ke, Fan Bai, Zihan Li, Yanbing Zhu, Chunjia Zhang, Yan Li, Zuliyaer Talifu, Yunzhu Pan, Wubo Liu, Xin Xu, Feng Gao, Degang Yang, Liangjie Du, Yan Yu, Jianjun Li

**Affiliations:** ^1^Shandong University, Jinan, Shandong, China; ^2^China Rehabilitation Research Center, Beijing Bo’ai Hospital, Beijing, China; ^3^University of Health and Rehabilitation Sciences, Qingdao, Shandong, China; ^4^China Rehabilitation Science Institute, Beijing, China; ^5^Beijing Key Laboratory of Neural Injury and Rehabilitation, Beijing, China; ^6^Center of Neural Injury and Repair, Beijing Institute for Brain Disorders, Beijing, China; ^7^School of Rehabilitation, Capital Medical University, Beijing, China; ^8^Beijing Clinical Research Institute, Beijing Friendship Hospital, Capital Medical University, Beijing, China

**Keywords:** spinal cord injury, phospholipase D, neuroinflammation, transcriptome sequencing analysis, protein microarray analysis

## Abstract

**Introduction:**

Spinal cord injury (SCI) is a severely disabling disease. Hyperactivation of neuroinflammation is one of the main pathophysiological features of secondary SCI, with phospholipid metabolism playing an important role in regulating inflammation. Phospholipase D (PLD), a critical lipid-signaling molecule, is known to be involved in various physiological processes, including the regulation of inflammation. Despite this knowledge, the specific role of PLD in SCI remains unclear.

**Methods:**

In this study, we constructed mouse models of SCI and administered PLD inhibitor (FIPI) treatment to investigate the efficacy of PLD. Additionally, transcriptome sequencing and protein microarray analysis of spinal cord tissues were conducted to further elucidate its mechanism of action.

**Results:**

The results showed that PLD expression increased after SCI, and inhibition of PLD significantly improved the locomotor ability, reduced glial scarring, and decreased the damage of spinal cord tissues in mice with SCI. Transcriptome sequencing analysis showed that inhibition of PLD altered gene expression in inflammation regulation. Subsequently, the protein microarray analysis of spinal cord tissues revealed variations in numerous inflammatory factors. Biosignature analysis pointed to an association with immunity, thus confirming the results obtained from transcriptome sequencing.

**Discussion:**

Collectively, these observations furnish compelling evidence supporting the anti-inflammatory effect of FIPI in the context of SCI, while also offering important insights into the PLD function which may be a potential therapeutic target for SCI.

## 1 Introduction

Spinal cord injury (SCI) is a severely disabling condition, which often leads to irreversible neurological impairment and an imposition of a substantial economic burden. The treatment of SCI remains one of the greatest challenges for basic science research and clinical medicine. Although several therapeutic approaches have been explored, the effectiveness has thus far been limited. The pathophysiology of SCI encompasses both primary and secondary injuries. The process of secondary SCI is intricate, involving a cascade of biochemical mechanisms such as neuroinflammation, neuronal apoptosis, demyelination, and glial response following the initial injury. These processes contribute to the exacerbation and extension of SCI, making it a complex phenomenon (Bramlett and Dietrich, 2007). Given the difficulty in avoiding primary injury, current therapeutic strategies for SCI primarily revolve around mitigating the effects of secondary injury.

The over-activation of neuroinflammation is a prominent pathophysiological characteristic of secondary injury. As observed above, the neuroinflammation mechanism is intricate, and activating various immune cells, secreting inflammatory factors, and the recruiting immune cells from the circulatory system to the site of SCI. Inhibiting the excessive activation of immune responses following SCI stands as a crucial therapeutic target, significantly influencing the functional recovery of the spinal cord (Anderson et al., 2016; Hellenbrand et al., 2021). Recent studies have revealed that various phospholipid metabolism-related molecules, such as phospholipase A, phospholipase C, and the related metabolite lysophosphatidic acid, play important roles in SCI. It has been shown that dysregulation of these phospholipid metabolisms can lead to excessive secondary inflammation and that these dysregulated phospholipids, if not broken down, may lead to the development of chronic inflammation (Fujita et al., 2018; David and Lopez-Vales, 2021).

Phospholipase D (PLD) is a vital lipid signaling molecule associated with several physiological activities such as cell proliferation, differentiation, membrane transport, and cytoskeleton assembly. It is the basis for many physiological processes (Frohman, 2015). The primary role of PLD is the hydrolysis of phosphatidylcholine into phosphatidic acid (PA) and choline, with PA serving as the primary functional agent of PLD (Bowling et al., 2021). Previous studies have highlighted the significant involvement of PLD in various central nervous system disorders, including drug addiction, cognitive impairment, and neurodegenerative diseases, which include Parkinson’s disease (Krishnan, 2016; Conde et al., 2018; McDermott et al., 2020). Notably, studies have also reported the involvement of PLD in the activation of macrophages and astrocytes during autoimmune encephalomyelitis (Ahn et al., 2001), Additionally, researchers have observed a gradual increase in PLD expression within the first week after SCI, with the majority of PLD-positive cells identified as activated macrophages and astrocytes (Jung et al., 2003), suggesting that PLD may be involved in inflammatory regulation and glial scar generation in SCI. However, a comprehensive understanding of the specific mechanisms underlying the role of PLD in spinal cord injury remains elusive.

We hypothesized that PLD is a potential therapeutic target for reducing secondary injury in SCI. 5-fluoro-2-indolyl des-chlorohalopemide (FIPI) is a PLD inhibitor that can effectively inhibit its activity with no significant side effects. Although it has low solubility in water, it can be dissolved in organic solvents (Su et al., 2009) and has been widely used as a PLD inhibitor in a variety of studies. In this study, we constructed mouse models of SCI and treated them with FIPI to evaluate its efficacy. Furthermore, we used transcriptome sequencing and protein tissue microneedle column analysis were used to explore the underlying mechanisms of action.

## 2 Materials and methods

### 2.1 Animals

The experimental protocol was approved by the Institutional Animal Care and Use Committee of Capital Medical University, Beijing, China. Adult female C57BL/6N (18–22 g) mice were purchased from the Experimental Animal Center of Capital Medical University (Beijing, China). The mice were housed in an environment maintained at 22 ± 2°C and 55 ± 10% humidity and were exposed to a 12-h light/dark cycle with *ad libitum* access to food and water.

### 2.2 Experimental design

Animals were randomly assigned to three groups: (1) the sham-operated group underwent only laminectomy without SCI; (2) the SCI group underwent SCI followed by immediate intraperitoneal injection of dimethyl sulfoxide (DMSO); and (3) the SCI+FIPI group was given 0.9 mg/kg body weight of FIPI (Sigma, HY-12807) by intraperitoneal injection immediately after SCI. The treatment was maintained until the mice were killed for further analysis.

FIPI was formulated according to previous literature (Su et al., 2009). FIPI was solubilized in DMSO at a concentration of 10 μM. Before administration, FIPI solution was further diluted to a working concentration of 0.5 μM in saline. A daily dose of 0.9 mg per kg of body weight was administered to the mice.

### 2.3 SCI modeling and tissue collection

The SCI modeling procedure was carried out as previously documented (Jing et al., 2021). Briefly, mice were fully anesthetized with 2% isoflurane, and the dorsal region was meticulously prepared by shaving and disinfecting. The vertebral plates covering the T10 segment were exposed, followed by their removal to expose the spinal cord. Subsequently, a controlled strike force of 70 kilodynes was applied to the spinal cord’s dorsal surface using the Infinite Horizons Impactor (Precision Systems & Instrumentation, Lexington, KY, USA). Following the injury, the surgical incision was closed layer by layer, and the mice were intraperitoneally injected with either FIPI or an equivalent volume of DMSO solution. The animal was then placed in a recovery chamber. Post-injury, the mice bladders were manually emptied twice daily.

For RNA and protein analyses, the mice were anesthetized via intraperitoneal injection of an appropriate dose of sodium pentobarbital. Subsequently, 1-cm-long segments of the spinal cord encompassing the injury epicenter were rapidly dissected, temporarily frozen in liquid nitrogen, and then stored at −80°C.

For histopathological and immunofluorescence examinations, mice were anesthetized with an appropriate dose of sodium pentobarbital via intraperitoneal injection. They were perfused transcardially with saline followed by paraformaldehyde. The spinal cords were quickly dissected and preserved in paraformaldehyde. Then, 3-μm-thick sections were prepared from paraffin-embedded tissues for hematoxylin and eosin (H&E) staining and immunofluorescence assessments.

### 2.4 Motor function assessment

Motor function in mice was evaluated using the Basso Mouse Scale (BMS), a scoring system previously described in the literature (Jing et al., 2021). Before the assessment, mice were placed in the evaluation environment for a 15-min acclimatization period. Subsequently, the hip-ankle movements of the lower limbs were carefully observed to assess paw postures and weight-bearing. Scores were assigned based on the observed outcomes. The scoring intervals were set at pre-modeling and at 3, 7, 14, 21, 28, 35, and 42 days post-modeling.

To evaluate mouse locomotion further, the open field test was conducted using CleverSys TopScan LITE. This system precisely tracked the mouse’s real-time position and monitored parameters such as speed and distance traveled. Mice were familiarized with the square open field for 15 min, after which the system recorded their movements within the area for 5 min. Subsequently, locomotor abilities were assessed based on the recorded data. The assessment was conducted 42 days after SCI.

### 2.5 Histologic analysis

Standard histological procedures were followed, which included the dehydration and paraffin embedding of spinal cord sections in both transverse and longitudinal orientations, with a section thickness of 3 μm. Subsequently, the sections underwent dewaxing and hydration and were subjected to routine hematoxylin and eosin staining.

Images at a magnification of 20 × were captured using the digital TissueFAXS imaging system. These images were then analyzed using TissueFAXS software (TissueGnostics, Vienna, Austria) to examine the structural characteristics within the SCI region.

### 2.6 Immunofluorescence

Tissue sections were subjected to deparaffinization using xylene and anhydrous ethanol. Antigen retrieval was performed using citrate buffer (pH 8), followed by rinsing with PBS. Subsequently, tissue sections were delineated using an immuno-histochemical pen, and a 10% bovine serum albumin (BSA) solution was applied for 30 min to block non-specific binding. Primary antibodies, suitably diluted, were then added and incubated at 4°C overnight. After rinsing away the primary antibodies with PBS, corresponding secondary antibodies were introduced and incubated for 50 min at room temperature. Following another wash with PBS, an anti-fluorescence quenching sealer containing DAPI (P0131, Beyotime, China) was applied. The sections were sealed, and digital images were acquired using the TissueFAXS imaging system. Subsequent analysis was carried out using TissueFAXS software (TissueGnostics, Vienna, Austria).

In our study, we demarcated the necrotic area by utilizing the SCI site and the medial edge of GFAP as reference boundaries. Subsequently, we quantified the extent of necrosis. The average fluorescence intensity of GFAP was measured by isolating a region of residual tissue located 1 mm from the periphery of necrotic SCI tissue. Furthermore, a region situated 3–5 mm from the injury core was analyzed to determine the average fluorescence intensity of MBP. Finally, we measured the average fluorescence intensity of NF200 within the spinal cord tissue region.

Primary and secondary antibodies used included: GFAP (1:100, CST, 80788); MBP (1:500, Servicebio, GB11226); NF200 (1:500, Servicebio, GB12144); and CY3 goat anti-rabbit IgG (1:500, Service-bio, GB21303).

### 2.7 RT-PCR

Total RNA was extracted from spinal cord tissues using the Total RNA Extraction Kit (DP419, Tengen) following the manufacturer’s instructions. The concentration and purity of the extracted RNA were assessed using a Nanodrop 2000 spectrophotometer (Thermo Fisher Scientific, Waltham, MA, USA). Subsequently, individual samples were aliquoted and diluted, and cDNA synthesis was accomplished using the cDNA First Strand Synthesis Kit (G3337, Servicebio, Wuhan, China).

Quantitative reverse transcription-polymerase chain reaction (qRT-PCR) was conducted with the qPCR premix (G3327, Servicebio, Wuhan, China) utilizing the following parameters: an initial denaturation at 95°C for 10 min, followed by 95 cycles of denaturation at 40°C for 15 and annealing/extension at 60°C for 60 s. The relative mRNA expression levels were determined employing the ΔΔCT method, with GAPDH as the reference gene for normalization. Each sample analysis was performed in triplicate. Additionally, Supplementary material includes the primer sequences used for PCR.

### 2.8 Transcriptome sequencing and analysis

The spinal cord tissues were collected from each group, and the sequencing method was performed according to the manufacturer’s standard protocol of Annoroad Gene Technology Co., Ltd, Beijing, China. The total RNA of the samples was first extracted and examined for the concentration and quality of the RNA. Then, the mRNA was enriched and purified with magnetic beads attached to oligo (dT), followed by the addition of Fragmentation Buer to split the mRNA into short fragments. This was used as a template, reverse transcribed using random primers to synthesize the first strand of cDNA, after which the second strand of cDNA was synthesized. The product was subjected to end repair, addition of base A, and addition of sequencing junction. The library preparation was completed by screening out the target fragments using magnetic beads and performing PCR amplification. After the library construction was completed, it was quantified and diluted to 1 ng/μL. Q-PCR was performed using a fluorescent quantitative PCR instrument (Bio-RAD CFX) to determine the quality of the library, and the quality-qualified library was sequenced using the Illumina platform.

The information analysis process uses the raw reads obtained from sequencing on the Illumina platform, and then through the process of removing low-quality sequences, removing splice contamination, and other processes to complete the data processing to obtain high-quality sequences (Clean Reads), according to which the expression amount is calculated and comparative analysis of the data is carried out.

Differential gene screening was mainly based on the criteria of fold change and *q*-value (Padj value, corrected *P*-value), and the genes that reached the criteria of log2 fold change ≥0.5 and *q* < 0.05 were regarded as significant differential genes (R language, version 4.2.3). Functional analysis was performed by the ClusterProfiler (4.7.1), such as Gene Ontology (GO) and Kyoto Encyclopedia of Genes and Genomes (KEGG).

### 2.9 Protein microarray analysis

The spinal cord tissues were collected from each group. A total of 200 cytokines were detected in mouse spinal cord tissues using the GSM-CAA-4000 microarray kit, and the assay method was performed according to the manufacturer’s standard protocols. Firstly, spinal cord tissue proteins were extracted, and the protein concentration was determined using BSA. Dry slide chips were prepared, and a sealing solution was added to close each microarray well. After aspirating the sealing solution, add an equal amount of sample to the Quantitative antibody chip well, and incubate at 4°C overnight. Thoroughly wash the slides with a chip washer, then dilute the detection antibody to the appropriate concentration and add it to the detection wells and incubate at room temperature for 2 h. Thoroughly wash again and add the prepared Cy3-streptavidin to the assay wells and incubate for 1 h at room temperature in the dark. Finally, the wells were thoroughly washed and the cy3 fluorescence signal was detected using an InnoScan 300 Microarray Scanner laser scanner (Innopsys, France). The microarray data were analyzed by the data analysis system accompanying GSM-CAA-4000, and the raw data were normalized according to the results of the internal reference to screen out the differential proteins with the criteria of |log2 fold change| > 0.263 and *q* < 0.05, and the screened differential proteins were subjected to biological analyses to determine their functions, including GO functional clustering analysis and KEGG pathway signaling pathway.

### 2.10 Statistical analysis

According to the statistical analysis for this study, it was conducted utilizing SPSS software (IBM Corporation, Armonk, NY, USA, version 26.0). All data were presented as mean ± standard deviation. In the process of statistical analysis, an initial assessment of normality was performed on each dataset. For comparisons between two groups, the independent samples *t*-test is employed for datasets exhibiting normal distribution. In cases involving multiple groups, one-way ANOVA was applied to normally distributed datasets. Non-normally distributed data were analyzed using non-parametric tests. Samples with values that deviate significantly from the rest of the sample were eliminated according to statistical methods. Statistical significance was considered at *P* < 0.05. The results were graphically represented using GraphPad Prism 8.0 software (GraphPad Software, Inc.) for visualization.

## 3 Results

### 3.1 PLD expression increases after SCI

**FIGURE 1:**
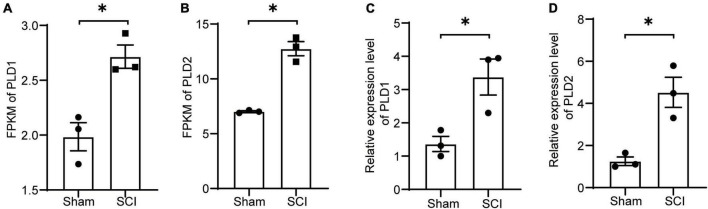
Changes in the expression levels of PLD after SCI. **(A,B)** Seven days post-SCI in mice, spinal cord tissues from the injured site were subjected to transcriptome sequencing analysis. The FPKM values for PLD1 and PLD2 are presented (*n* = 3). **(C,D)** Seven days post-SCI in mice, injured spinal cord tissues were collected, and the relative expression levels of PLD1 and PLD2 were assessed using RT-PCR (*n* = 3). *P < 0.05 (Student’s *t*-test). FPKM, Fragments per Kilobase Million. PLD, Phospholipase D.

Samples were collected at 7 days after SCI for transcriptome sequencing to investigate the changes in PLD expression after SCI. The transcriptome results suggested an increase in the expression levels of PLD1 and PLD2 in the SCI group compared to the sham control group (Figures 1A, B).

To validate these findings, spinal cord samples at the 7-day time point were subjected to PCR analysis. The PCR results for PLD1 and PLD2 exhibited a consistent trend of change observed in the transcriptome data, confirming the accuracy of our transcriptome analysis (Figures 1C, D). These results indicated the potential involvement of PLD in the progression of SCI in mice.

### 3.2 Inhibition of PLD significantly improves locomotor activity in mice after SCI

**FIGURE 2:**
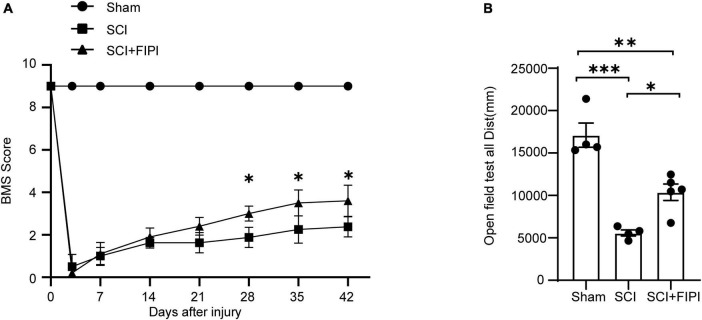
Inhibitory effects of PLD on the motor function of SCI mice. **(A)** BMS scores at various time points post-spinal cord injury (SCI) in three groups of mice (*n* = 4–5). *Indicates significant differences between SCI and SCI+FIPI groups (one-way ANOVA). **(B)** Open field test was conducted 42 days post-spinal cord injury for the three groups of mice, demonstrating differences in motor abilities. Shown here is the total distance moved by the mice within 5 min (*n* = 4–5). *P < 0.05, **P < 0.01, ***P < 0.001 (one-way ANOVA). BMS, Basso Mouse Scale.

To investigate the effect of PLD on the prognosis of SCI, we conducted a study in which FIPI was administered to mice following spinal cord contusion. The motor capabilities of the mice in each group were evaluated using the BMS score and the open-field test (Figure 2).

The post-operative scores of the SCI and SCI+FIPI groups were both 0, with gradual increases in the score in the subsequent weeks. Notably, mice in the SCI+FIPI group displayed an improvement in BMS scores compared to the SCI group at 3 weeks after injury. Significant enhancement in BMS scores was evident at 4 weeks, and the difference remained at 6 weeks (Figure 2A).

The open-field test found that at 6 weeks after SCI, mice in the SCI+FIPI group demonstrated significantly greater total distance covered and movement speed than the SCI group (Figure 2B). These data collectively indicated that FIPI treatment yields a substantial enhancement in motor function after SCI.

### 3.3 Inhibition of PLD protects spinal cord tissue in SCI mice

**FIGURE 3:**
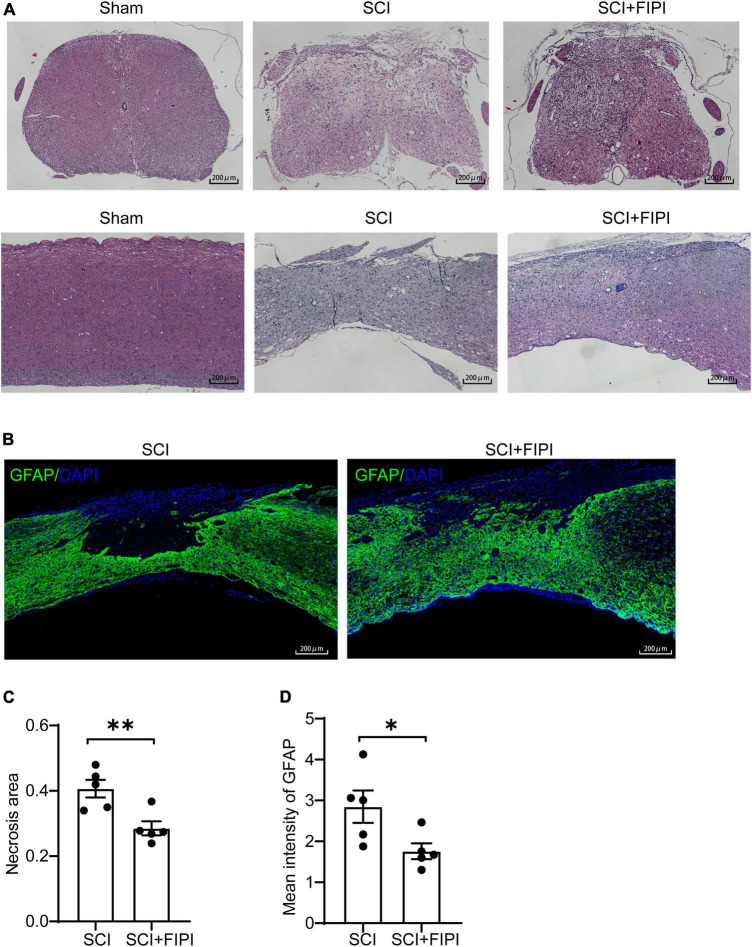
The therapeutic effect of FIPI treatment on spinal cord tissue in SCI mice. **(A)** HE staining of transverse and longitudinal sections of the spinal cord in different groups 6 weeks post-SCI. (20X, scale bar = 200 μm). **(B)** Immunostaining of GFAP (FITC, 20X, scale bar = 200 μm, *n* = 5). **(C)** Quantification of the necrotic area within the spinal cord sections for SCI and SCI+FIPI groups. **(D)** Quantification of the average fluorescence intensity of GFAP within residual spinal cord tissue within 1 mm from the injury core (*n* = 5). *P < 0.05, **P < 0.01 (Student’s *t*-test), HE, hematoxylin-eosin; GFAP, glial fibrillary acidic protein.

To investigate the effect of FIPI treatment on the microstructure of the injured spinal cord, we conducted sections on samples taken 6 weeks after SCI. Hematoxylin and eosin (HE) staining revealed that the extent of spinal cord tissue atrophy in the SCI+FIPI group was significantly less than that observed in the SCI group (Figure 3A). GFAP serves as a marker for the activation of astrocytes, shedding light on the presence of neuroglial scarring. Immunofluorescence staining for GFAP indicated a reduction in the necrotic area in the FIPI-treated group compared to the SCI group (Figures 3B, C).

These findings suggested that FIPI treatment can potentially attenuate the injury, preserving more tissue integrity. Moreover, we quantitatively analyzed the fluorescence intensity of GFAP and found that the average fluorescence intensity in the FIPI-treated group was lower than that in the SCI group, signifying a reduction in the formation of glial scarring (Figure 3D).

### 3.4 Inhibition of PLD protects neural conduction structures in SCI mice

**FIGURE 4:**
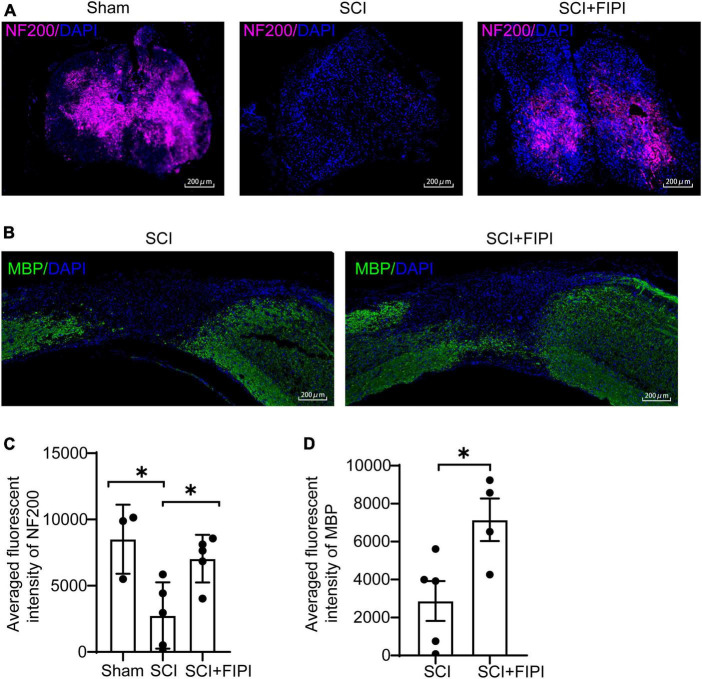
The therapeutic effect of FIPI treatment on neural conduction structures in SCI mice. **(A)** Tissue samples were collected 42 days post-spinal cord injury, and paraffin sections were prepared for transverse sections with immunofluorescent staining for NF200 (CY5, 20X, scale bar = 200 μm). **(B)** Longitudinal sections of paraffin-embedded tissue were prepared for MBP immunofluorescent staining (FITC, 20X, scale bar = 200 μm) **(C)** Quantification of the average fluorescence intensity of NF200 within spinal cord tissue (one-way ANOVA, *n* = 3–5). **(D)** Quantification of MBP within 3–5 mm from the injured core (*n* = 4–5). *P < 0.05 (Student’s *t*-test). NF200, neurofilament-200. MBP, myelin basic protein.

To elucidate the anatomical foundation of locomotor function, we conducted immunofluorescent staining of MBP and NF200 on sections of the spinal cord. NF200 is an essential structural protein in neurons that contributes to the stability and integrity of neuronal axons. We observed that the fluorescence intensity of NF200 was notably higher in the FIPI-treated group than the SCI group, indicating the presence of more nerve fibers owing to FIPI treatment (Figures 4A, C). MBP is a key component of the myelin sheath which plays a crucial role in the structure and function of the nervous system. The MBP intensity in the FIPI-treated group was significantly greater than that in the SCI group, suggesting that FIPI treatment mitigated post-SCI demyelination (Figures 4B, D).

Collectively, these results suggested that FIPI treatment can reduce tissue atrophy and preserve the nerve conduction structures in the spinal cords of injured mice.

### 3.5 Transcriptome sequencing analysis of spinal cord tissue one-week post-SCI

**FIGURE 5:**
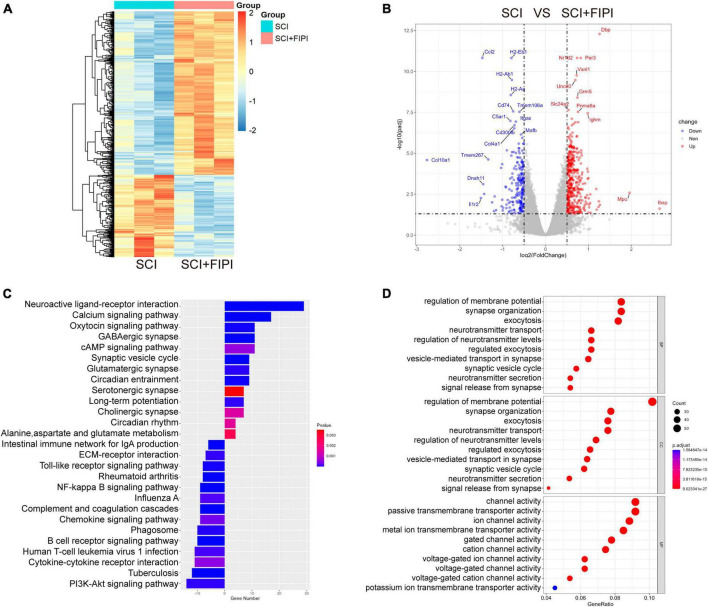
Transcriptome sequencing analysis of spinal cord tissue one-week post-SCI (*n* = 3). **(A,B)** Heatmap and Volcano plot illustrating differential genes in the SCI+FIPI group compared to the SCI group (log2 Fold change ≥0.5 and *q* < 0.05). **(C)** KEGG analysis results based on differential genes in the SCI+FIPI group compared to the SCI group. **(D)** Presentation of the top 10 entries in the GO analysis, encompassing Biological Process, Molecular Function, and Cellular Component categories. KEGG, Kyoto Encyclopedia of Genes and Genomes; GO, Gene Ontology.

Exporing the mechanism of FIPI treatment, spinal cord tissue samples were collected one week after SCI for mRNA transcriptome sequencing. The transcriptional profiles of the FIPI treatment group were compared with those of the SCI group, resulting in the identification of 616 significantly differentially expressed genes (fold change ≥0.5; *P* < 0.05). Out of these, 410 genes were upregulated and 206 were downregulated. These findings were visually represented in the heatmap and volcano plot (Figures 5A, B).

Subsequently, GO analysis was performed based on the different genes. This analysis included cellular component, biological process, and molecular function. The cellular component analysis revealed a strong association of the differential genes with the cellular membrane, consistent with the known distribution of PLD. The biological process analysis indicated correlations with cellular membrane activities, including extracellular secretion and vesicular transport. Molecular function analysis suggested differences in the activity of various membrane ion channels. The GO analysis indicated that FIPI treatment affects a spectrum of cellular membrane-related activities (Figure 5D).

Furthermore, the KEGG pathway analysis was conducted on the selected genes. This analysis uncovered significant enrichment in pathways such as the cAMP signaling pathway, PI3K-Akt signaling pathway, and MAPK signaling pathway, as shown in Figure 5C. Several pathways were strongly associated with immune responses.

### 3.6 FIPI treatment alleviates inflammatory responses in the spinal cord tissue of mice after SCI

**FIGURE 6:**
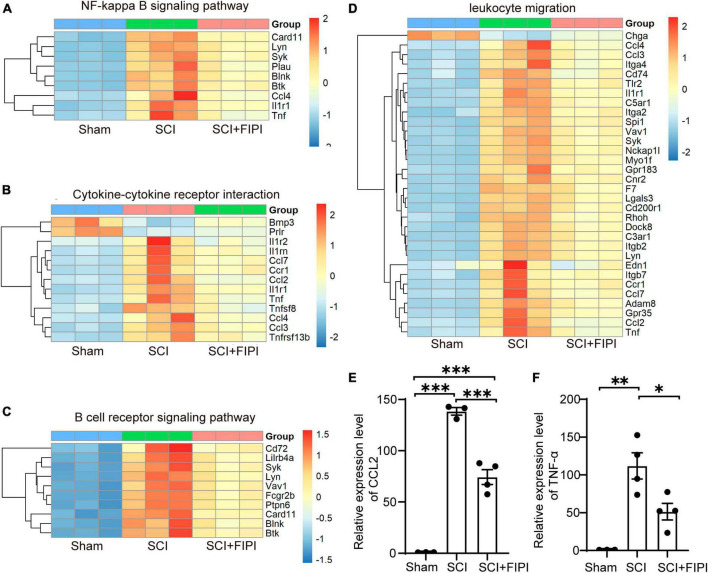
Presentation of inflammation-related results in transcriptome analysis (*n* = 3). **(A–C)** Enriched inflammatory pathways identified in KEGG analysis. **(D)** In GO analysis, differential genes are shown to play a role in leukocyte migration. **(E,F)** The expressions of CCL2 and TNF-α detected by RT-PCR were assessed in spinal cord tissues 7 days after SCI. *P < 0.05, **P < 0.01, ***P < 0.001 (one-way ANOVA).

Differential gene expression analysis in our transcriptome results revealed variations in inflammation-related signaling pathways and functional analyses between the SCI and SCI+FIPI groups. In the GO analysis of molecular function based on the differential genes, differences were observed in leukocyte migration (Figure 6D). Additionally, KEGG analysis identified numerous pathways associated with inflammation, including cytokine-cytokine receptor interaction, B cell receptor signaling pathway, and NF-kappa B signaling pathway (Figures 6A–C). Therefore, we postulate that inhibition of PLD could reduce the extent of inflammation within the spinal cord tissue of SCI mice, consistent with its documented anti-inflammatory effects in other pathological contexts.

Among the plethora of differentially expressed genes in the transcriptome dataset, we identified classical inflammatory factors CCL2 and TNF-α (*P* < 0.05). These factors were subsequently selected for PCR validation to confirm the accuracy of the transcriptome results. The results of PCR demonstrated significant down-regulation of both CCL2 and TNF-α in the FIPI-treated group compared to the SCI group, in accordance with the trend observed in the transcriptome dataset (Figures 6E, F).

In summary, PLD plays a crucial role in the inflammatory response process of SCI.

### 3.7 Protein microarray analysis of spinal cord tissue

**FIGURE 7:**
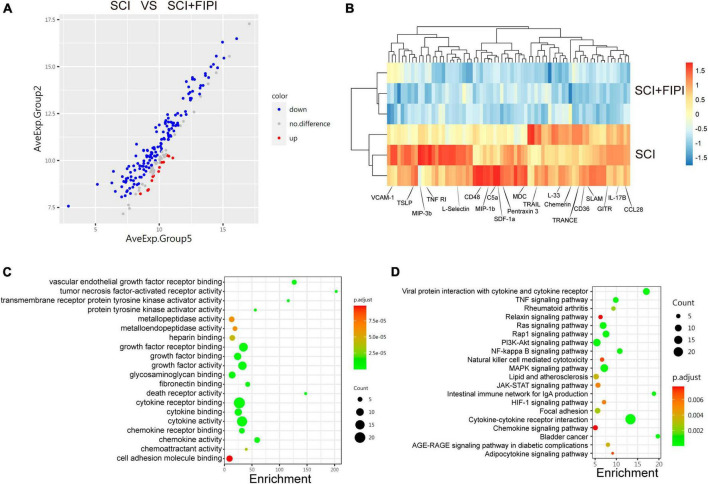
Protein microarray analysis of spinal cord tissue one-week post-SCI (*n* = 3). **(A)** Volcano plot displayed differentially expressed cytokines which were defined as those with log2 Fold change > 0.263 and *q* < 0.05. **(B)** Heatmap illustrating some of the differential genes in the SCI and SCI+FIPI groups. **(C)** GO analysis of molecular function based on the differential genes. **(D)** KEGG analysis based on the differential genes.

To further investigate the role of PLD in SCI, we conducted protein microarray analysis of spinal cord tissue collected from sham, SCI, and SCI+FIPI groups 7 days post-SCI. This test enabled us to assess the levels of 200 different cytokines semi-quantitatively, and finally, differently express 71 cytokines between the SCI and SCI+FIPI groups (|log2 fold change| > 0.263 and *q* < 0.05). To visually represent these findings, we generated a volcano plot and heat map based on the identified differentially expressed proteins (Figures 7A, B), which showed that various inflammatory factors exhibited significant down-regulation.

Subsequently, we subjected the 71 identified differential factors to GO analysis. The analysis of biological processes revealed that these differential proteins were associated with various activities, including leukocyte migration and chemoregulation. Furthermore, molecular function analysis indicated a close relationship with inflammatory functions, including tumor necrosis factor-activated receptor activity and cytokine activity (Figure 7C).

Moreover, KEGG enrichment analysis enable us to identify variations in several inflammation-related pathways, including the TNF-signaling pathway and the NF-kappa B signaling pathway. These outcomes align with the outcomes of transcriptome sequencing, providing additional support for the anti-inflammatory effect of FIPI on SCI at the protein level (Figure 7D).

## 4 Discussion

There is increasing evidence to support the involvement of dysregulated phospholipid metabolism in secondary injury associated with SCI. PLD is a key player in phospholipid metabolism, a process underlying numerous pathophysiologies (Frohman, 2015). In this study, we investigated the expression levels of PLD in mice at 7 days after SCI. The findings revealed a gradual increase in PLD expression following SCI, suggesting its potential involvement in secondary injury processes post-SCI. It is worth noting that previous research has also reported a similar gradual increase in PLD expression within 4 days post-SCI (Jung et al., 2003), which is consistent with our study results.

We utilized FIPI, a potent inhibitor of PLD, to treat mice suffering from SCI. Our findings revealed that inhibiting PLD activity not only diminished the extent of SCI but also led to a reduction in glial scarring. It also aided in the preservation of the nerve conduction structure. Ultimately, these positive outcomes facilitated the recovery of motor function in mice afflicted with SCI.

To investigate the underlying mechanisms, we conducted the transcriptome sequencing analysis, which revealed a marked elevation in inflammation within the injured spinal cord of mice, reaffirming the involvement of inflammation in the secondary damage of SCI. The result also showed significant variations in the expression of numerous genes between the SCI+FIPI group and the SCI group. Subsequent analysis of these differentially expressed genes demonstrated that FIPI treatment effectively reduced the level of inflammation at the injury site of the spinal cord. To confirm the reliability of the transcriptome results, we assessed the expression of inflammatory markers, CCL2 and TNF-α, using RT-PCR, and observed results consistent with the trends in the transcriptome data. This provided robust evidence for the reliability of our conclusions from the RNA level.

Given the inherent short lifespan of mRNA (Baudrimont et al., 2017) and the intricate regulatory mechanisms involved in mRNA translation into proteins (Sahu et al., 2023), discrepancies exist between the quantities of RNA and proteins. Considering the disparities observed in the transcriptome results, we employed protein microarray technology to detect changes in proteins. This method is capable of detecting up to 200 cytokines, and we identified 71 differentially expressed proteins by using this approach. Subsequently, we conducted an analysis of the differential proteins, and the results were consistent with our previous findings. This reinforced the notion that FIPI treatment could mitigate inflammation secondary to SCI, and PLD inhibition could be an effective treatment approach for SCI.

Phospholipase D is an important lipid signaling molecule, associated with a series of physiological activities including cell proliferation, differentiation, membrane transport, and cytoskeleton assembly (Krishnan et al., 2011). So far, six PLD isoforms (PLD1–PLD6) have been identified, with PLD1 and PLD2 emerging as the most prominent isoforms. Both are ubiquitously expressed in virtually all tissues and cell lines and are found in neurons and glial cells in the central nervous system (McDermott et al., 2020). Within animal tissues, PLDs predominantly localize to the cytosolic membrane and perinuclear compartments including the Golgi apparatus, endoplasmic reticulum, or late vesicles (Krzystanek et al., 2020), in alignment with their functional roles.

The main function of PLDs is to hydrolyze phosphatidylcholine into phosphatidic acid (PA) and choline. PA constitutes a pivotal component of the PLD-dependent second messenger system (Frohman, 2015). Notably, PA interacts with a spectrum of downstream regulatory proteins, including various kinases, phosphatases, G-protein regulatory factors, and phosphodiesterases (Mendez-Gomez et al., 2018). There are four major pathways for cellular PA generation in mammals (Tanguy et al., 2018), but the 36:1 PA species produced by PLD is not identical to the other pathways, and the functions of the different species of PA vary, so the pathways are not complete substitutes for each other (Sim et al., 2020). Furthermore, PA can undergo conversion into two other biologically active lipid molecules, diacylglycerol (DAG) and lysophosphatidic acid (LPA), indirectly participating in an array of regulatory processes (Krishnan et al., 2011).

Phospholipase D exhibits upregulation in response to diverse cellular stresses, including hypoxia and nutrient deprivation (Ganesan et al., 2020). Previous studies have demonstrated the involvement of PLD in immune responses in various diseases, including neurofunctional and degenerative disorders (Gobel et al., 2014; Mateos et al., 2014; Schonberger et al., 2014; Bermudez et al., 2019). Moreover, PLD’s metabolite, PA, has been implicated in the regulation of some metabolic pathways that are involved in immunomodulation, such as the Raf, mTOR, and the Hippo pathway (Han et al., 2018; Kattan et al., 2022). These signaling pathways may also influence the regulation of inflammatory responses within the central nervous system, encompassing the recruitment of granulocytes, macrophages, and microglia, as well as the production of pro-inflammatory cytokines and chemokines (Ding and Chen, 2022). In SCI and brain injury, the level of LPA, which is converted from PA, also rises rapidly, and LPA is also involved in the activation of immune cells, leading to inflammation, demyelination, and other deleterious effects in the central nervous system (David and Lopez-Vales, 2021). The differential genes identified in our present study also hint at an association with a diverse range of immune cells.

Prior investigations in a variety of diseases have also demonstrated the ability of PLD and its metabolite, PA, to influence actin cytoskeleton dynamics and proteins related to cell migration, which could orchestrate the migration and phagocytosis of granulocytes and macrophages (Ali et al., 2013; Bohdanowicz et al., 2013; Cho and Han, 2017; Gomez-Cambronero and Ganesan, 2018; Urbahn et al., 2018). Therefore, in the current study, we hypothesized that FIPI could inhibit the migration of immune cells such as neutrophils and macrophages to the site of SCI. In line with this hypothesis, our study identified differentially expressed factors closely associated with macrophages, such as macrophage-derived chemokines (MDCs) and CD36. Moreover, our KEGG analysis of the transcriptome sequencing unveiled enrichment in signaling pathways related to cell migration, phagosome, cell adhesion molecules, and calcium signaling pathway, among others. Consequently, we hypothesized that in the context of SCI, PLD may exert its regulatory effect on macrophage migration to the injury site of the spinal cord, ultimately mitigating secondary inflammation of SCI.

The activation of glial cells plays an important role in central nervous system inflammation (Zhu et al., 2015; Linnerbauer et al., 2020). Notably, a previous study observed increased PLD expression in activated macrophages and astrocytes following SCI (Jung et al., 2003), suggesting the involvement of PLD in astroglial regulation. In our investigation, we observed that the level of GFAP immunofluorescence in the FIPI treatment group was notably reduced compared to that in the SCI group. These differences may have arisen from FIPI’s direct modulation of astrocytes and the indirect attenuation of astroglial activation by inhibiting other immune cells that attenuate local inflammation.

Furthermore, T cells and B cells have significant roles in SCI (Donnelly and Popovich, 2008; David and Lopez-Vales, 2021). In our current study, we identified variations in lymphocyte-related inflammatory factors, including thymic stromal lymphopoietin (TSLP), L-selectin, and CD48. These differences in inflammatory factors may arise from the regulatory effects of PLDon multiple immune cell types. In future research, our research aim to gather further direct evidence to enhance our comprehensive understanding of the specific mechanisms governing PLD’s influence on diverse immune cell types.

Phospholipase D plays a crucial role in a wide array of diseases, and significant therapeutic effects have been achieved by inhibiting PLD in certain disease models. In a study of ischemic stroke, selective inhibition of PLD1 substantially reduced infarct area and cerebral edema in mice, leading to improved neurological function scores. Simultaneous inhibition of both PLD1 and PLD2 resulted in enhanced therapeutic effects (Lien et al., 2020). PLD inhibition has also been proven to reduce the inflammatory response in a broad spectrum of diseases. In septicemia, inhibiting PLD enzymatic activity reduced PLD-mediated cell adhesion and migration, resulting in lowered TNF-α levels in septic mice plasma and reduced migration of leukocytes and platelets to the lungs (Urbahn et al., 2018). In the realm of tumors, PLD not only affects the migration, proliferation, and invasion of tumor cells, but also participates in tumor-related inflammatory reactions through pathways such as NF-κB (Cho and Han, 2017). Several *in vitro* experimental studies have shown that the inflammatory response can be attenuated by inhibiting PLD. Researchers have found that the level of LPS-induced inflammation in retinal pigment epithelium (RPE) can be alleviated via PLD inhibition, and confirmed that PLD promotes the inflammatory response by regulating ERK1/2 and COX-2 (Mateos et al., 2014). In another study based on the oxygen-glucose deprivation and reoxygenation (OGD/R) model, inhibiting PLD significantly suppressed the production of pro-inflammatory factors and the activation of NLRP3 inflammasomes in RPE cells, reducing cell death caused by OGD/R-induced ferroptosis (Park et al., 2023). Another study found that PLD also regulates the level of inflammation in the RPE through protein kinase C. Inhibition of PLD protects the RPE from lipopolysaccharide-induced injury (Tenconi et al., 2016). Similarly, in a study of chronic rhinosinusitis (CRS), inhibition of PLD was found to reduce PKC phosphorylation and thus reduce inflammation in extracted human mucosa-derived fibroblast cells. Furthermore, it was reported that this process does not involve the activation of phospholipase C, but only in relation to the action of PLD (Tsai et al., 2021). In summary, PLD widely participates in the body’s inflammatory response and is therefore considered a potential regulatory target.

FIPI, as an inhibitor of PLD, has been used in numerous studies to demonstrate, its broad prospects in various medical fields. FIPI produces significant effects at low concentrations, with no noteworthy side effects. It has shown greater potency in *in vivo* assays (0.5 nM IC50) than in standard *in vitro* assays (25 nM IC50) (Su et al., 2009). Due to its hydrophobicity results in low solubility in water, FIPI powder needs to be dissolved in an organic solvent for optimal delivery. FIPI has limited bioavailability (18%) and relatively short half-life (5.5 h) (Ganesan et al., 2015). Additionally, FIPI compounds have >30 off target activities, which may react with numerous biogenic amine receptors (Stieglitz, 2018). The potential tolerance to FIPI is also a consideration, as prolonged PLD inhibition may trigger compensatory activation of alternative pathways for PA production, thus attenuating the inhibitory effect on PLD (Frohman, 2015). To remedy these limitations of FIPI, researchers have been continuously optimizing strategies to develop new inhibitors based on pharmacological and pharmacokinetic characteristics. This study initially explored the feasibility of using FIPI to inhibit PLD for treating SCI, and it is hoped that future efforts can lead to the development of more effective inhibitors for clinical application through changes in formulation or structure.

## 5 Conclusion

Phospholipase D holds a critical position in the context of SCI. By inhibiting PLD function following SCI, it could reduce tissue inflammation, diminish glial scarring, maintain the integrity of nerve conduction structures, and ultimately improve motor function in mice. Moving forward, our research will persist in exploring the role of PLD within diverse immune cell types, aiming to investigate the precise mechanisms through which PLD is implicated in the inflammatory response.

## Data availability statement

The data presented in this study has been deposited in the NCBI GEO repository, accession number: GSE249615; https://www.ncbi.nlm.nih.gov/geo/query/acc.cgi?acc=GSE249615.

## Ethics statement

The animal study was approved by the Animal Care and Use Committee of Capital Medical University (Ethics Batch No. AEEI-2023-326). The study was conducted in accordance with the local legislation and institutional requirements.

## Author contributions

HK: Conceptualization, Data curation, Formal Analysis, Investigation, Project administration, Writing – original draft. FB: Conceptualization, Data curation, Formal Analysis, Writing – review and editing. ZL: Investigation, Methodology, Software, Writing – original draft. YZ: Conceptualization, Investigation, Resources, Writing – review and editing. CZ: Methodology, Software, Visualization, Writing – review and editing. YL: Validation, Writing – review and editing. ZT: Validation, Writing – original draft. YP: Formal Analysis, Writing – original draft. WL: Data curation, Writing – original draft. XX: Validation, Writing – original draft. FG: Writing – review and editing, Project administration. DY: Writing – review and editing, Visualization. LD: Writing – review and editing, Data curation. YY: Data curation, Writing – review and editing, Conceptualization, Formal Analysis, Funding acquisition, Supervision. JL: Conceptualization, Formal Analysis, Funding acquisition, Supervision, Writing – review and editing.
