# Supplementary material for: Inhibition of phospholipase D promotes neurological function recovery and reduces neuroinflammation after spinal cord injury in mice
Source: Front Cell Neurosci. 2024 Mar 20;18:1352630. doi: 10.3389/fncel.2024.1352630 (PMC10987874; doi:10.3389/fncel.2024.1352630)
Supplement: Supplementary file 1 [file Table_1.DOCX]

Supplementary Material

Table1. The primer sequences used for PCR

| Name | Primer sequences (5’-3’) |
| --- | --- |
| PLD1 | F-TGCTGAGATACCGCTGCAACTTAG  R-CTTGGCACCCTTGAGGTCGATG |
| PLD2 | F-TTCAGCCTCTGAAAGCACACC  R-GTAAAGTCACCATGCGTCAAGC |
| CCL2 | F-CAGGTCCCTGTCATGCTTCT  R-GTCAGCACAGACCTCTCTCT |
| TNF-α | F-CCGATGGGTTGTACCTTGTC  R-AGATAGCAAATCGGCTGACG |
| GAPDH | F-CCTCGTCCCGTAGACAAAATG  R-TGAGGTCAATGAAGGGGTCGT |
